# Impact of the COVID-19 Pandemic and Lockdown on Cancer Diagnoses Using Swiss Cantonal Cancer Registry Data

**DOI:** 10.3390/cancers16193381

**Published:** 2024-10-03

**Authors:** Flurina Suter, Miriam Wanner, Dominik Menges, Andreas Wicki, Dimitri Korol, Sabine Rohrmann

**Affiliations:** 1 Division of Chronic Disease Epidemiology, Department of Epidemiology, Epidemiology, Biostatistics and Prevention Institute (EBPI), University of Zurich, 8001 Zurich, Switzerland; flurina.suter2@uzh.ch (F.S.);; 2 Cancer Registry Zurich, Zug, Schaffhausen and Schwyz, Institute of Pathology and Molecular Pathology, University Hospital Zurich, 8006 Zurich, Switzerland; 3 Department of Medical Oncology and Hematology, Faculty of Medicine, University Hospital Zurich, 8091 Zurich, Switzerland

**Keywords:** coronavirus disease 2019, directly age-standardized cancer incidence rate, regression analysis, Switzerland, cancer registry

## Abstract

**Simple Summary:**

Limited knowledge exists on the effects of the COVID-19 pandemic and the pandemic lockdown on cancer detection and diagnoses in Switzerland. Population-based Swiss cantonal cancer registry data of the cantons of Zurich and Zug from 2018/19 to 2021 were used. Differences in cancer diagnoses were analyzed descriptively and using multivariate regression models. A decrease in all-cancer incidence was observed in 2020 compared to 2018/19, mainly during the lockdown phase in April. Meanwhile, higher all-cancer incidence rates were observed in 2021 compared to 2018/19. The impact in Switzerland was less severe than in other countries. Our findings may inform the decisions of policymakers and the public health system during future pandemics.

**Abstract:**

**Background/Objectives:** This study aims to investigate differences in cancer diagnosis based on absolute case numbers and age-standardized incidence rate ratios (IRRs) in the pre-Coronavirus disease 2019 (COVID-19) years (2018/19) and the first two years of the COVID-19 pandemic (2020, 2021) in two Swiss cantons. **Methods**: Data of the Swiss cantonal cancer registry of the cantons of Zurich (ZH) and Zug (ZG) were used to descriptively investigate differences in annual and monthly absolute numbers regarding all-cancer and the five most common cancer types. Directly age-standardized monthly incidence rates (IRs) were calculated. Multivariate Quasipoisson regression models were fitted to determine the IRRs with 95% confidence intervals (95% CI). **Results**: Annual absolute numbers of all investigated cancers were similar in 2018/19, 2020, and 2021, except for prostate cancer (increase of 20.8% in 2021 compared to 2018/19). In 2020, there were generally more cancer diagnoses in January and February followed by a decrease in April and May. Compared to the pre-COVID-19 period, lower IRs were observed in 2020 for all-cancer (IRR = 0.96 [95% CI 0.96, 0.97]) and female breast cancer (0.92 [0.89, 0.96]), whereas higher IRs were observed in 2021 for all-cancer (1.02 [1.02, 1.02]) and prostate cancer (1.23 [1.18, 1.28]). **Conclusions**: Cancer detection and diagnoses decreased during the first year of the pandemic, especially during the most stringent lockdown phase in April. The findings of this study may inform the decisions of policymakers and public health system during future pandemics.

## 1. Introduction

In December 2019, the severe acute respiratory syndrome coronavirus 2 (SARS-CoV-2) was first detected in Wuhan, China [1,2]. Shortly after, SARS-CoV-2 spread worldwide, causing the coronavirus disease 2019 (COVID-19) pandemic [1,2]. Due to its potential severe effects on human health, the health systems, and its associated high morbidity and mortality [1,2,3], COVID-19 became a highly prioritized global public health problem. From March 2020, Switzerland implemented various public health measures on a national level, aiming to decrease the number of COVID-19 infections [4]. As part of these measures, a national lockdown of public life from 17 March to 26 April 2020 was mandated, leading to a decrease in SARS-CoV-2 transmission [4]. In Switzerland, the last federal measures against the COVID-19 pandemic, namely the mandatory isolation for infected people and the mask mandate in public transport and healthcare facilities, were repealed on 1 April 2022 [5].

As a consequence of the COVID-19 pandemic and particularly during lockdowns, diagnosis and treatment of other severe diseases, such as cancer, may have been neglected. In Swiss cantons with organized cancer screening programs, a strong decrease in conducted mammograms and colonoscopies was observed during the lockdown phase in March 2020 [6]. As in Switzerland, the majority of European countries stopped or slowed down their cancer screening programs due to the COVID-19 pandemic [7,8].

As a result, a decrease in all-cancer diagnoses during the pandemic was reported for several countries [9,10,11,12]. A decreasing trend was also reported for specific cancer types, such as colorectal cancer [8,9,13], lung cancer [9,13], skin melanoma [14], female breast cancer [9,13], prostate cancer [15], head and neck cancer [16], and pancreatic, gastric, and oesophageal cancer [13].

The manifold impacts of the COVID-19 pandemic and lockdown on cancer are rarely investigated in Switzerland. To the best of our knowledge, there is one other study based on data of several Swiss University Hospitals [14], but none based on population-based cancer registry data. Therefore, the aim of this study was to use population-based cantonal cancer registry data to investigate the effect of the COVID-19 pandemic and especially the lockdown on cancer diagnosis in Switzerland.

## 2. Materials and Methods

The structure of this study followed the Strengthening the Reporting of Observational Studies in Epidemiology (STROBE) guidelines [17].

### 2.1. Cancer Registry and Study Population

For this study, patients diagnosed with cancer between 2018 and 2021 and registered at the Cantonal Cancer Registry of the cantons of Zurich (ZH), Zug (ZG), Schaffhausen (SH), and Schwyz (SZ) (CRZZSS) were included. The canton of ZH started registration in 1980 and the canton of ZG joined in 2011. For the cantons of SH and SZ, which started registration in the year 2020, no data on the pre-COVID-19 years (2018/19) were available, and therefore their data were excluded from this study. Up to now, no cantonal cancer screening programs are implemented in the cantons of ZH and ZG [6]. In 2020, a new Swiss law on cancer registration was implemented at a national level [18]. The law obligates all private and public medical institutions to report all cancer cases to the respective cantonal cancer registry [18]. The CRZZSS collects detailed information on cancer patients, including personal data such as sex and age, and medical data regarding the cancer, such as date of diagnosis, type and extension of cancer, as well as treatment information. Cancer cases diagnosed in 2018 and 2019 were combined into a pre-COVID-19 period to minimize random annual fluctuations and thereby receive a more reliable estimate. Meanwhile, the incidence years 2020 and 2021 were kept as separate years due to different COVID-19 measures being implemented during these years. The CRZZSS uses the international classification of diseases (10th revision) (ICD-10) [19] code for the documentation of cancer cases. In this study, registered patients with a malignant cancer (except ICD-10 C44) or a benign brain cancer (ICD-10: D32–33, D43) were used to investigate changes in diagnoses for all-cancer. Additionally, the five most common cancer sites in Switzerland were examined separately: colorectal cancer (ICD-10 C18–C20), lung cancer (ICD-10 C34), skin melanoma (ICD-10 C43), female breast cancer (ICD-10 C50), and prostate cancer (ICD-10 C61).

### 2.2. Statistical Analyses

The baseline characteristics of the investigated cancer cases stratified by cancer type were summarized descriptively. The absolute number of cancer diagnoses was calculated on an annual level and monthly level, overall and stratified by canton (ZH, ZG) and sex (males, females). In combination with the monthly stringency of the public health measures in Switzerland based on the Oxford COVID-19 Government Response Tracker (OxCGRT) [20] the absolute number of all-cancer cases from 2020 to 2021 were visualized. The OxCGRT ranges from 0 to 100 with higher numbers indicating a more stringent lockdown phase [20]. In total, four different OxCGRT indices were calculated for over 180 countries on a daily basis from 1 January 2020 onwards [20]. The OxCGRT indices are based on 19 policy indicators covering impacts on health systems, economic responses, containments and closures, and other responses. Of those 19 indicators, 9 (focusing on containment and closure policies, also referred to as lockdown policies) were used to establish one of the four stringency indices [20]. The latter OxCGRT stringency index was used in the current study by calculating the mean index value per incidence month and year. The absolute and relative difference in annual and monthly case numbers between the pre-COVID-19 (2018/19) period and the year 2020 and 2021 were determined. Directly age-standardized monthly incidence rates by incidence year (2018/19, 2020, 2021) were calculated overall and by canton (ZH, ZG) and sex (males, females). The corresponding 95% confidence intervals (95% CI) were calculated using the method by Fay and Feuer [21]. The age-standardized monthly incidence rates are visualized in Figures. In addition, the latter all-cancer rates were visualized for 2018 and 2019 separately to detect possible differences among the two pre-COVID-19 years. Quasipoisson regression models adjusted for canton (ZH, ZG), sex (males, females), incidence year (2018/19, 2020, 2021), and incidence month (January–December) were fitted to determine incidence rate ratios (IRRs) with their corresponding 95% CI. In the Quasipoisson regression models, the month of January was set as reference month, because it was least affected by the COVID-19 lockdown measures based on the OxCGRT stringency index.

As a sensitivity analysis, an interaction term between incidence year and month was added to the Quasipoisson regression model for all-cancer, colorectal cancer, female breast cancer, and prostate cancer, i.e., high incidence cancers that are usually detected by screening.

The software R (version 4.3.1 [22]) was used to conduct the analyses. As the statistical significance level, a two-sided *p*-value of 0.05 was defined for all analyses.

## 3. Results

The baseline characteristics of the study population stratified by cancer subtype are displayed in Table 1. The annual absolute number of cancer cases was similar among the investigated incidence years (average 2018–2019 = 8586; 2020 = 8427; 2021 = 9015). Among all diagnosed cancers included in this study, 92.5% were registered in the canton of ZH and 7.5% in the canton of ZG. The age at incidence was similar across the investigated cancer types, with a median age ranging from 69.0 to 71.0 years of age, except for skin melanoma and female breast cancer with a lower median age at incidence of 66.0 and 63.0 years, respectively. An equal sex distribution was seen for colorectal cancer (48.7% females, 51.3% males). For all-cancer, lung cancer, and skin melanoma, slightly more males (52.9%, 55.8% and 54.3%) than females (47.1%, 44.2% and 45.7%) were affected. The annual distribution of cancer types was similar among the incidence years, except for a slight increase in prostate cancer in 2021 (28.6%) compared to 2018/19.

Stratifying the annual absolute all-cancer case numbers by incidence month, canton, and sex, similar trends can be observed among the two sexes, although males had generally higher monthly case numbers. The canton of ZG showed more fluctuation due to smaller absolute case numbers (Figures S1 and S2).

**Figure 1:**
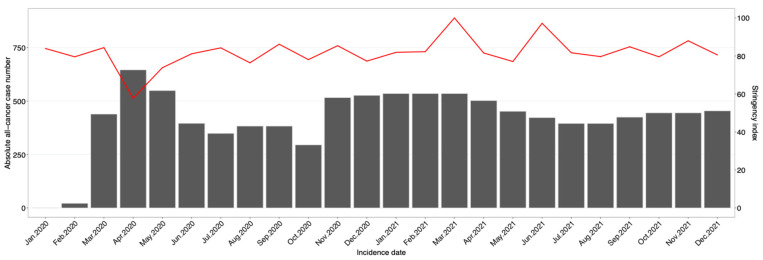
The absolute number of monthly all-cancer cases by incidence year in the Swiss cantons of Zurich and Zug combined (red line) and the monthly stringency index of the public health measures in Switzerland (gray bars) for the years 2020 and 2021.

The absolute numbers of monthly cancer diagnoses by incidence year in the Swiss cantons of ZH and ZG in addition to the pandemic measures stringency index are visualized in Figure 1. The most stringent period based on the OxCGRT stringency index was seen in April 2020 due to the lockdown, where, simultaneously, the largest drop in cancer diagnoses was observed. As the stringency index decreased from May 2020 to July 2020, the number of diagnoses increased simultaneously. From July 2020 onwards, the previously observed negative association between the number of diagnoses and the stringency index almost vanished, leading to peaks in the number of diagnoses that did not continue to correlate with a low stringency index and vice versa.

In Table 2, the absolute and relative annual and monthly incidence counts by cancer type and period for both cantons combined are listed. Furthermore, the absolute and relative difference in case numbers between the pre-COVID-19 period and the incidence years 2020 and 2021 are determined. For all-cancer, colorectal cancer, lung cancer, skin melanoma, and female breast cancer, the annual number of cases were similar between the years. However, the monthly numbers revealed stronger variations between the incidence years. In 2020, there were, in general, more cancer diagnoses in January and February (increase of up to 52.7% [lung cancer in February]), followed by a decrease in April and May (decrease of up to −57.6% [skin melanoma in April]) compared to 2018/19. In the second half of the year 2020, the variations between the years were smaller, and more variation among the cancer types was observed. In 2021, no clear pattern in monthly diagnoses was seen across the investigated cancer types.

For prostate cancer in the year 2020, there was a drop in new diagnoses only in April and May (−37.5% and −6.9%), whereas during all other months, the number of diagnoses was the same or higher than in the pre-COVID-19 period. In 2021, compared to the pre-COVID-19 period, a strong increase in prostate cancer diagnoses, 20.8%, was observed. In every month of 2021, an increase between 1.7% and 40.2% of prostate cancer diagnoses compared to the respective numbers in 2018/19 was observed.

The estimates and 95% CIs of the Quasipoisson regression model when investigating the age-standardized monthly cancer incidence rate while adjusting for canton, incidence year, incidence month, and sex (all-cancer, skin melanoma, colorectal cancer, and lung cancer, respectively) are displayed in Table 3. Females had lower cancer IRs than males for all cancer types with IRRs ranging from 0.73 [95% CI: 0.70, 0.76] for lung cancer to 0.83 [0.82, 0.83] for all-cancer. The canton of ZG had lower IRs than ZH for all-cancer (IRR = 0.96 [0.95, 0.96]) and prostate cancer (IRR = 0.92 [0.89, 0.96]), whereas higher IRs were seen for colorectal cancer (IRR = 1.30 [1.26, 1.34]), lung cancer (IRR = 1.13 [1.08, 1.18]), skin melanoma (IRR = 1.46 [1.41, 1.52]), and female breast cancer (IRR = 1.15 [1.11, 1.19]). In comparison to the pre-COVID-19 period, for ZH and ZG combined, lower IRs were observed for all-cancer (IRR = 0.96 [0.96, 0.97]) and female breast cancer (IRR = 0.92 [0.89, 0.96]) in 2020, but not for other cancer types. Meanwhile, higher IRs were observed for all-cancer (IRR = 1.02 [1.02, 1.02]) and prostate cancer (IRR = 1.23 [1.18, 1.28]) in 2021, but not for other cancer types. When investigating the IRRs for each month compared to the reference month (January), all-cancer and every cancer type, except lung cancer, showed evidence for lower IRs in April, with IRRs ranging from 0.73 [0.67,0.79] for prostate cancer to 0.90 [0.84,0.95] for colorectal cancer and 0.90 [0.89,0.90] for all-cancer. However, no further trend among the cancer types was observed.

**Figure 2:**
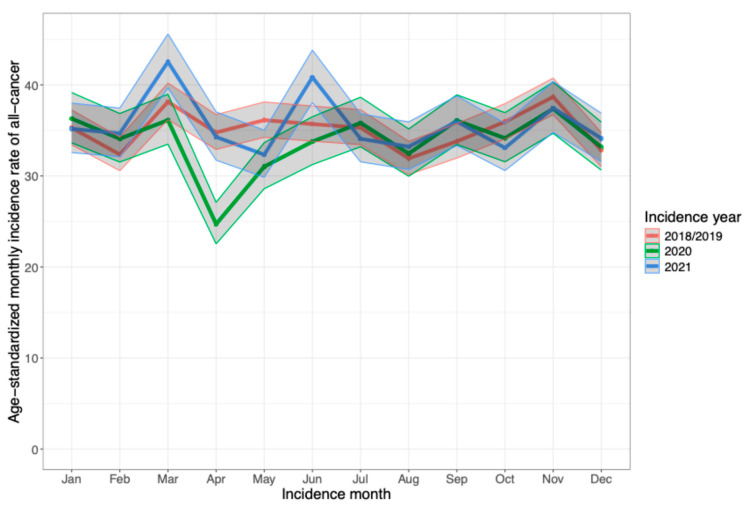
Age-standardized incidence rates with 95% confidence intervals of monthly cancer diagnoses for all-cancer in the Swiss cantons of Zurich and Zug stratified by incidence year.

In Figure 2, the age-standardized IRs and 95% CI by incidence month and year are visualized for all-cancer. Generally, the IRs were similar among the years (indicated by overlapping 95% CIs), except in April 2020, when the IRs were markedly lower in comparison to 2018/19 and 2021. In May 2020, the IRs were still noticeably lower than in the pre-COVID-19 period. The same pattern observed for all-cancer was also seen for the five cancer types, but was less pronounced (Figures S3–S7). Figure S8 shows the all-cancer age-standardized IRs for the two pre-COVID-19 years separately, indicating small fluctuations between the two years.

In the sensitivity analyses (adding an interaction term between the year and month of incidence to the Quasipoisson model), lower IRs were seen in April compared to January for all-cancer, female breast cancer, and prostate cancer, but not for colorectal cancer (Table S1). Focusing on the interaction terms between April and the incidence years, no significant differences in the IRs were seen for female breast cancer in April 2020, but were in April 2021 (IRR = 1.25 [1.08,1.43]) compared to April 2018/19. For colorectal and prostate cancer, lower IRs were observed in April 2020 (IRR = 0.49 [0.42; 0.57]; IRR = 0.48 [0.40; 0.57]), but there was no significant difference in the IRs in April 2021, whereas for all-cancer, lower IRs were seen in April 2020 (IRR = 0.70 [0.69,0.71]) as well as in April 2021 (IRR = 0.98 [0.97; 1.00]) in comparison to April 2018/19. Significant estimates and interaction terms were seen for other months than April as well, showing additional variation in cancer diagnoses between the months (Table S1).

## 4. Discussion

Based on the population-based cantonal cancer registry data of the cantons of ZH and ZG, the absolute numbers of all-cancer, colorectal cancer, lung cancer, skin melanoma, and female breast cancer diagnoses were similar between the pre-COVID-19 period and the first two years of the COVID-19 pandemic. In contrast, a strong increase in absolute case numbers was seen for prostate cancer in 2021 compared to pre-pandemic numbers. From April to May 2020, when the pandemic measures’ stringency was highest in Switzerland, lower absolute numbers of cancer diagnoses were seen for all-cancer and all five investigated cancer types. In comparison to the pre-COVID-19 period, lower IRs were observed for all-cancer and female breast cancer in 2020, whereas higher IRs were observed for all-cancer and prostate cancer in 2021. The latter-observed IRs of all-cancer in 2020 and 2021 might reflect a catch-up process in 2021 of missed cancer diagnoses in 2020.

Only a slight decrease in all-cancer cases was observed in 2020 compared to 2018/19 in this study, indicating a less severe impact of the COVID-19 pandemic than observed in several other countries [7,10]. Based on annual data, 6% fewer all-cancer diagnoses were made in Belgium in 2020 compared to the pre-COVID year, 2019, [23] and in Finland, there were 4.3% (*n* = 1600) fewer all-cancer diagnoses in 2020 than expected based on pre-pandemic incidence [24]. In 2021, compared to 2018/19, our study observed higher annual absolute numbers of all-cancer cases. The higher absolute number in 2021 might be due to the new law on cancer registration in Switzerland, enforcing the reporting of new cancer cases to the respective cantonal cancer registries [18]. We can speculate whether, without the influence of the COVID-19 lockdown, the overall absolute numbers of documented cancer diagnoses would have shown an increase in 2020 and 2021. Therefore, the new law might have counteracted the effect of the COVID-19 lockdown, especially in the year 2020.

As in our study, several previous studies have investigated the annual absolute numbers of diagnoses of specific cancer types before and during the COVID-19 pandemic, reporting decreases during the pandemic of up to 50% [9,25,26]. A systematic review by Angelini et al. included 61 studies on cancer diagnosis and diagnostic tests [10]. When comparing the lockdown period (January to October 2020) with the pre-COVID period, strong decreases for skin melanoma (−22.6%), breast (−19.8%), genito-urinary (−28.9%), prostate (−26.2%), gastro-intestinal (−25.1%), colorectum (−24.1%), and skin cancer (−27.6%) were seen [10]. In contrast, our study found rather small decreases in absolute case numbers between 2018/19 and 2020, and even a slight increase for lung cancer. In 2021, compared to the pre-COVID-19 years, an increase in cancer diagnoses was observed for all-cancer and all investigated cancer types, except for lung cancer. The observed slight increase in lung cancer diagnoses in 2020 compared to pre-COVID-19 may be due to the increased number of conducted lung examinations related to COVID-19. Prostate cancer was the only cancer type revealing an increase in absolute numbers of cancer diagnoses in both COVID-19 years, 2020 and 2021, compared to the pre-COVID-19 period. In comparison, a systematic review including 40 studies mainly from the US reported a decrease in prostate cancer diagnoses during the COVID-19 pandemic, ranging from −4.1% to −71.7%, in combination with a reduction in prostate cancer screening rates, ranging from nearly 0% to −78% [15]. There are several possible reasons that could have influenced the observed higher number of prostate cancer diagnoses in the current study. First, there exists no cancer screening program in the cantons of ZH and ZG that could have been interrupted, causing fewer diagnoses as observed for several cancer types in other European countries [7], such as Belgium [23] and Poland [27]. Secondly, the screening recommendations for the Swiss population are adapted regularly, and the procedures to detect prostate cancer increase in variety and accuracy [28]. Lastly, the Swiss population steadily increases in age, reflected by a high and increasing life expectancy [29], and the immigration rate in Switzerland belongs to the highest ones in Europe [30]. The latter two aspects lead to more people in the high-risk population groups and, therefore, more potential cases.

In contrast to the annual absolute numbers, the monthly absolute numbers in our study are in accordance with the trends observed in other countries. In our study, lower absolute numbers of cancer diagnoses were observed from April to May 2020, coinciding with the strict lockdown period and with the highest OxCGRT stringency index values. During the lockdown in Switzerland, healthcare institutions had to delay all non-urgent procedures and elderly people were advised to stay home [5], possibly being the main cause for the observed decrease in cancer diagnoses during April and May, 2020. Additional reasons for the above-mentioned decline in cancer diagnoses could be the increase in acutely ill and, therefore, hospitalized patients related to COVID-19, which could have limited the capacity of the Swiss healthcare system [31,32] and the observed excess mortality during the lockdown in spring 2020 [33]. Fewer cancer diagnoses during a COVID-19 wave and lockdown period compared to the pre-pandemic period were observed in several other countries as well, such as Belgium [23], Germany [8,34,35], Finland [24], England [36], and the US [13]. A Swiss study focusing on skin melanoma using data of several Swiss University Hospitals is in line with the results of our study, reporting a decrease in skin melanoma cancer diagnoses during the lockdown phase compared to the pre- and post-lockdown periods [14]. However, in our study, the observed negative association between the number of diagnoses and the stringency index during the lockdown phase disappeared from July 2020 onwards.

In our study, lower IRs were observed for all-cancer and female breast cancer in 2020 and higher IRs for all-cancer and prostate cancer in 2021 compared to the pre-COVID-19 period. The numbers align with the standardized incidence ratios (SIR) for all-cancer, which are calculated by using the indirect method observed by Ribes et al. in Catalonia, Spain, after two pandemic years (0.88 [0.87, 0.89]) [12] and by Trojanowski et al. in 2020 in western Poland (males: 0.80 [0.78, 0.81]; females: 0.83 [0.81, 0.85]) [11]. The latter study reported lower SIRs for female breast cancer in 2020 as well (0.88 [0.84, 0.92]) [11]. The study by Greene et al. calculated IRRs for the year 2020 compared to 2019 and observed lower IRRs for female breast cancer (0.81 [0.76, 0.86]) [9]. In contrast to our study, previous studies found lower rate ratios in 2020 compared to 2019 for further cancer types, such as colorectal [9,11], lung cancer [9,36], and skin cancer [11], possibly indicating that the Swiss healthcare system might have been less affected by the pandemic and lockdown measures than other countries.

One of the limitations of the current study is the simultaneous occurrence of the COVID-19 pandemic in Switzerland and the introduction of the new Swiss national law on cancer registration in 2020. Therefore, their effects on cancer incidence in the years 2020 and 2021 cannot be separated. Another limitation is the inclusion of only two Swiss cantons, even though the canton of ZH is the largest canton with respect to the number of inhabitants. Observed trends for the cantons of ZH and ZG might not be generalizable to the whole country. Nevertheless, the current study has multiple strengths. To the best of our knowledge, the current study is the first one using population-based cantonal cancer registry data of Switzerland to investigate the effects of the lockdown due to the COVID-19 pandemic on cancer diagnoses. Even though the new national law on cancer registration can be seen as a limitation factor, it is also a strength of the current study, ensuring complete and high-quality data for the incidence years 2020 and 2021. In contrast to comparable previous studies, our study included two pre-COVID-19 incidence years (2018, 2019) and the first two years of the COVID-19 pandemic (2020, 2021), instead of comparing two single incidence years or even only two monthly periods with each other. Therefore, the data of our study includes more representative pre-pandemic and pandemic data and can capture the long-term and not only the initial effects of the COVID-19 measures in Switzerland on cancer diagnoses. The findings of our study were based on data of regions with no implemented cancer screening programs, representing a previously hardly investigated context. Furthermore, our study investigated cancer diagnosis trends in general, but also the trends among the five most common cancer types in Switzerland. Lastly, by calculating age-standardized IRRs, our results are directly comparable to those of previous studies.

## 5. Conclusions

In conclusion, cancer detection and diagnoses decreased during the first COVID-19 year (2020), especially during the most stringent lockdown phase in April. However, the effects were less severe and for a shorter period than observed in other countries, indicating that the Swiss healthcare system was able to continue providing high-quality care throughout the pandemic. The findings of the current study can inform the future decisions of policymakers and the public health system during a future SARS-CoV-2 wave or other future pandemics. Future population-based studies are needed to investigate the manifold effects of the COVID-19 lockdown on cancer and other severe diseases, also including further regions of Switzerland.

## Figures and Tables

**Table 1:** Characteristics of the investigated cancer cases in the Swiss cantons of Zurich and Zug from 2018 to 2021 combined.

Variables	All-Cancer ^1^	Colorectal Cancer ^1^	Lung Cancer ^1^	Skin Melanoma ^1^	Female Breast Cancer ^1^	Prostate Cancer ^1^
*n*	34,614	3291	3360	2984	4887	5474
Sex, *n* (%)						
Males	18,317 (52.9)	1688 (51.3)	1874 (55.8)	1620 (54.3)	0 (0)	5474 (100)
Females	16,297 (47.1)	1603 (48.7)	1486 (44.2)	1364 (45.7)	4887 (100)	0 (0)
Age at Incidence (median [IQR])	69.0	71.0	71.0	66.0	63.0	70.0
Canton, *n* (%)	[58.0, 77.0]	[60.0, 80.0]	[63.0, 77.0]	[52.0, 77.0]	[51.0, 74.0]	[64.0, 76.0]
Zurich (ZH)	32,017 (92.5)	3018 (91.7)	3123 (92.9)	2705 (90.7)	4484 (91.8)	5074 (92.7)
Zug (ZG)	2597 (7.5)	273 (8.3)	237 (7.1)	279 (9.3)	403 (8.2)	400 (7.3)
Incidence Year, *n* (%)						
2018–2019	17,172 (49.6)	1657 (50.3)	1686 (50.2)	1478 (49.5)	2462 (50.4)	2591 (47.3)
2020	8427 (24.3)	786 (23.9)	848 (25.2)	719 (24.1)	1161 (23.8)	1318 (24.1)
2021	9015 (26.0)	848 (25.8)	826 (24.6)	787 (26.4)	1264 (25.9)	1565 (28.6)

^1^ Cancer cases were defined using the tenth revision of the international classification of diseases (ICD-10): all-cancer: all malignant cancers (except C44) and benign brain cancer (ICD-10: D32–33, D43); colorectal cancer: ICD-10 C18–C20; lung cancer: ICD-10 C34; skin melanoma: ICD-10 C43; female breast cancer: ICD-10 C50; prostate cancer: ICD-10 C61 [19].

**Table 2:** Absolute and relative difference in annual and monthly incidence counts from 2018/19 to 2021 in the Swiss cantons of Zurich and Zug for all-cancer, colorectal cancer, lung cancer, skin melanoma, female breast cancer, and prostate cancer.

	**All-Cancer ^2^, 34,614**					**Colorectal Cancer ^2^, 3291**				
	**2018–2019** **(mean)**	**2020**	**Difference ^1^ (%)** **2020 vs. 2018–2019**	**2021**	**Difference ^1^ (%)** **2021 vs. 2018–2019**	**2018–2019** **(mean)**	**2020**	**Difference ^1^ (%)** **2020 vs. 2018–2019**	**2021**	**Difference ^1^ (%)** **2021 vs. 2018–2019**
Annual Incidence	8586	8427	−159 (−1.9)	9015	429 (5.0)	829	786	−43 (−5.2)	848	19 (2.3)
Monthly Incidence										
January	724	746	22 (3.0)	728	4 (0.6)	65	74	9 (13.9)	67	2 (3.1)
February	659	707	48 (7.3)	731	72 (10.9)	67	75	8 (11.9)	66	−1 (−1.5)
March	772	750	−22 (−2.9)	889	117 (15.2)	77	67	−10 (−13.0)	96	19 (24.9)
April	708	513	−195 (−27.5)	725	17 (2.4)	67	43	−24 (−35.8)	65	−2 (−3.0)
May	739	656	−83 (−11.2)	685	−54 (−7.3)	63	71	8 (12.7)	62	−1 (−1.6)
June	733	721	−12 (−1.6)	864	131 (17.9)	74	54	−20 (−27.0)	103	29 (39.2)
July	730	749	19 (2.6)	726	−4 (−0.6)	80	76	−4 (−5.0)	74	−6 (−7.5)
August	655	679	24 (3.7)	708	53 (8.1)	69	64	−5 (−7.3)	63	−6 (−8.7)
September	684	766	82 (12.0)	754	70 (10.2)	71	83	12 (16.9)	68	−3 (−4.2)
October	731	694	−37 (−5.1)	707	−24 (−3.3)	64	62	−2 (−3.1)	59	−5 (−7.8)
November	788	759	−29 (−3.7)	782	−6 (−0.8)	70	60	−10 (−14.3)	68	−2 (−2.9)
December	666	687	21 (3.2)	716	50 (7.5)	63	57	−6 (−9.5)	57	−6 (−9.5)
	**Lung Cancer ^2^, 3360**					**Skin Melanoma ^2^, 2984**				
	**2018–2019** **(mean)**	**2020**	**Difference ^1^ (%)** **2020 vs. 2018–2019**	**2021**	**Difference ^1^ (%)** **2021 vs. 2018–2019**	**2018–2019** **(mean)**	**2020**	**Difference ^1^ (%)** **2020 vs. 2018–2019**	**2021**	**Difference ^1^ (%)** **2021 vs. 2018–2019**
Annual Incidence	843	848	5 (0.6)	826	−17 (−2.0)	739	719	−20 (−2.7)	787	48 (6.5)
Monthly Incidence										
January	66	67	1 (1.5)	62	−4 (−6.1)	56	65	9 (16.1)	63	7 (12.5)
February	55	84	29 (52.7)	62	7 (12.7)	58	65	7 (12.1)	64	6 (10.3)
March	71	75	4 (5.6)	85	14 (19.7)	53	59	6 (11.3)	67	14 (26.4)
April	79	65	−14 (−17.7)	68	−11 (−13.9)	59	25	−34 (−57.6)	69	10 (17.0)
May	68	65	−3 (−4.4)	65	−3 (−4.4)	59	51	−8 (−13.6)	51	−8 (−13.6)
June	80	77	−3 (−3.8)	85	5 (6.3)	66	78	12 (18.2)	62	−4 (6.1)
July	79	65	−14 (−17.7)	68	−11 (−13.9)	64	64	0 (0.0)	67	3 (4.7)
August	69	76	7 (10.1)	66	−3 (−4.4)	55	66	11 (20.0)	66	11 (20.0)
September	59	69	10 (17.0)	61	2 (3.4)	72	69	−3 (−4.2)	63	−9 (−12.5)
October	70	73	3 (4.3)	63	−7 (−10.0)	67	55	−12 (−17.9)	67	0 (0.0)
November	73	74	1 (1.4)	63	−10 (−13.7)	78	73	−5 (−6.4)	84	6 (7.7)
December	77	58	−19 (−24.7)	78	1 (1.3)	56	49	−7 (−12.5)	64	8 (14.3)
	**Female Breast Cancer ^2^, 4887**					**Prostate Cancer ^2^, 5474**				
	**2018–2019** **(mean)**	**2020**	**Difference ^1^ (%)** **2020 vs. 2018–2019**	**2021**	**Difference ^1^ (%)** **2021 vs. 2018–2019**	**2018–2019** **(mean)**	**2020**	**Difference ^1^ (%)** **2020 vs. 2018–2019**	**2021**	**Difference ^1^ (%)** **2021 vs. 2018–2019**
Annual Incidence	1231	1161	−70 (−5.7)	1264	33 (2.7)	1296	1318	22 (1.7)	1565	269 (20.8)
Monthly Incidence										
January	109	101	−8 (−7.3)	101	−8 (−7.3)	128	130	2 (1.6)	152	24 (18.8)
February	82	97	15 (18.3)	112	30 (36.6)	105	107	2 (1.9)	128	23 (21.9)
March	111	95	−16 (−14.4)	119	8 (7.2)	126	129	3 (2.4)	161	35 (27.8)
April	97	67	−30 (−30.9)	101	4 (4.1)	104	65	−39 (−37.5)	126	22 (21.2)
May	112	80	−32 (−28.6)	90	−22 (−19.6)	116	108	−8 (−6.9)	118	2 (1.7)
June	100	110	10 (10.0)	116	16 (16.0)	114	115	1 (0.9)	155	41 (36.0)
July	105	111	6 (5.7)	101	−4 (−3.8)	88	112	24 (27.3)	105	17 (19.3)
August	86	84	−2 (−2.3)	88	2 (2.3)	100	100	0 (0.0)	131	31 (31.0)
September	108	98	−10 (−9.3)	120	12 (11.1)	95	98	3 (3.2)	100	5 (5.3)
October	121	106	−15 (−12.4)	93	−28 (−23.1)	92	95	3 (3.3)	111	19 (20.7)
November	111	104	−7 (−6.3)	126	15 (13.5)	132	138	6 (4.6)	142	10 (7.6)
December	92	108	16 (17.4)	97	5 (5.4)	97	121	24 (24.7)	136	39 (40.2)

^1^ The difference is calculated as the absolute and relative difference in incidence counts between the average number of the two reference years (2018–2019), rounded to the nearest digit, and the years 2020 and 2021, respectively. ^2^ Cancer cases were defined using the tenth revision of the international classification of diseases (ICD-10): all-cancer: all malignant cancers (except C44) and benign brain cancer (ICD-10: D32–33, D43); colorectal cancer: ICD-10 C18–C20; lung cancer: ICD-10 C34; skin melanoma: ICD-10 C43; female breast cancer: ICD-10 C50; prostate cancer: ICD-10 C61 [19].

**Table 3:** Quasipoisson regression model estimates of age-standardized monthly incidence rates of all-cancer and cancer subtype diagnoses in the Swiss cantons of Zurich and Zug from 2018/19 to 2021.

	**All-Cancer ^1,2^ (*n* = 34,614)**		**Colorectal Cancer ^1,2^ (*n* = 3291)**		**Lung Cancer ^1,2^ (*n* = 3360)**	
	**Estimate (IRR)**	**95% CI**	**Estimate (IRR)**	**95% CI**	**Estimate (IRR)**	**95% CI**
Sex						
Male (ref.)	1.00	-	1.00	-	1.00	-
Female	0.83	0.82–0.83	0.82	0.80–0.84	0.73	0.70–0.76
Canton						
Zurich (ref.)	1.00	-	1.00	-	1.00	-
Zug	0.96	0.95–0.96	1.30	1.26–1.34	1.13	1.08–1.18
Incidence Year						
2018–2019 (ref.)	1.00	-	1.00	-	1.00	-
2020	0.96	0.96–0.97	0.99	0.96–1.02	1.02	0.98–1.07
2021	1.02	1.02–1.02	1.02	0.99–1.05	1.03	0.98–1.07
Incidence month						
January (ref.)	1.00	-	1.00	-	1.00	-
February	0.94	0.93–0.94	1.04	0.98–1.10	0.95	0.87–1.05
March	1.09	1.08–1.10	1.20	1.14–1.27	1.10	1.00–1.20
April	0.90	0.89–0.90	0.90	0.84–0.95	0.97	0.89–1.07
May	0.94	0.94–0.95	0.95	0.89–1.01	0.89	0.81–0.98
June	1.03	1.02–1.03	1.10	1.04–1.16	1.02	0.93–1.12
July	0.99	0.98–1.00	1.07	1.02–1.14	1.09	0.99–1.19
August	0.91	0.91–0.92	0.95	0.89–1.00	0.98	0.89–1.07
September	0.99	0.98–1.00	1.04	0.98–1.10	0.89	0.80–0.98
October	0.97	0.96–0.97	0.91	0.86–0.97	0.90	0.82–0.99
November	1.07	1.06–1.08	1.02	0.96–1.08	0.95	0.87–1.04
December	0.94	0.93–0.95	0.94	0.89–1.00	1.03	0.94–1.12
	**Skin Melanoma ^1,2^ (*n* = 2984)**		**Female Breast Cancer ^1,3^ (*n* = 4887)**		**Prostate Cancer ^1,3^ (*n* = 5474)**	
	**Estimate (IRR)**	**95% CI**	**Estimate (IRR)**	**95% CI**	**Estimate (IRR)**	**95% CI**
Sex						
Male (ref.)	1.00	-	-	-	-	-
Female	0.82	0.80–0.85	-	-	-	-
Canton						
Zurich (ref.)	1.00	-	1.00	-	1.00	-
Zug	1.46	1.41–1.52	1.15	1.11–1.19	0.92	0.89–0.96
Incidence Year						
2018–2019 (ref.)	1.00	-	1.00	-	1.00	-
2020	0.98	0.95–1.02	0.92	0.89–0.96	1.00	0.95–1.04
2021	1.02	0.98–1.06	1.00	0.96–1.03	1.23	1.18–1.28
Incidence month						
January (ref.)	1.00	-	1.00	-	1.00	-
February	0.99	0.90–1.07	1.04	0.96–1.12	0.76	0.70–0.83
March	0.94	0.87–1.02	1.03	0.96–1.11	1.04	0.96–1.12
April	0.89	0.82–0.97	0.85	0.79–0.92	0.73	0.67–0.79
May	0.88	0.81–0.96	0.98	0.91–1.05	0.82	0.76–0.89
June	1.07	0.99–1.16	1.01	0.94–1.09	0.95	0.88–1.02
July	1.03	0.95–1.12	1.05	0.98–1.13	0.65	0.60–0.71
August	1.09	1.01–1.18	0.79	0.73–0.85	0.74	0.69–0.81
September	1.11	1.03–1.20	0.99	0.92–1.07	0.72	0.66–0.78
October	1.01	0.93–1.09	1.05	0.98–1.13	0.67	0.62–0.73
November	1.34	1.25–1.45	1.15	1.07–1.24	0.93	0.87–1.01
December	1.00	0.93–1.09	0.95	0.88–1.02	0.78	0.72–0.85

IRR = Incidence rate ratio; CI = Confidence Interval. ^1^ Cancer cases were defined using the 10th revision of the international classification of diseases (ICD-10): All-cancer: all malignant cancers (except C44) and benign brain cancer (ICD-10: D32–33, D43); colorectal cancer: ICD-10 C18-C20; lung cancer: ICD-10 C34; skin melanoma: ICD-10 C43; female breast cancer: ICD-10 C50; prostate cancer: ICD-10 C61 [19]. ^2^ The Quasipoisson regression model was adjusted for canton (Zurich, Zug), sex (males, females), incidence year (2018–2021), and incidence month (January-December). ^3^ The Quasipoisson regression model was adjusted for canton (Zurich, Zug), incidence year (2018–2021), and incidence month (January–December).

## Data Availability

The datasets generated during and/or analyzed during the current study are not publicly available since they are stored with personal identifiers, but are available from the corresponding author upon reasonable request.

## Supplementary Materials

The following supporting information can be downloaded at: https://www.mdpi.com/article/10.3390/cancers16193381/s1, Reference 1: World Health Organization. ICD-10 version: 2019 - International statistical classification of diseases and related health problems 10th revision. 2019. Accessed 18 November 2022. https://icd.who.int/browse10/2019/en; Figure S1: Absolute number of all-cancer cases stratified by incidence year, incidence month, and sex of the canton of Zurich from 2018/19 to 2021; Figure S2: Absolute number of all-cancer cases stratified by incidence year, incidence month, and sex of the canton of Zug from 2018/19 to 2021; Figure S3: Age-standardized incidence rates with 95% confidence intervals of monthly cancer diagnoses for colorectal cancer in the Swiss cantons of Zurich and Zug stratified by incidence year; Figure S4: Age-standardized incidence rates with 95% confidence intervals of monthly cancer diagnoses for lung cancer in the Swiss cantons of Zurich and Zug stratified by incidence year; Figure S5: Age-standardized incidence rates with 95% confidence intervals of monthly cancer diagnoses for skin melanoma in the Swiss cantons of Zurich and Zug stratified by incidence year; Figure S6: Age-standardized incidence rates with 95% confidence intervals of monthly cancer diagnoses for female breast cancer in the Swiss cantons of Zurich and Zug stratified by incidence year; Figure S7: Age-standardized incidence rates with 95% confidence intervals of monthly cancer diagnoses for prostate cancer in the Swiss cantons of Zurich and Zug stratified by incidence year; Figure S8: Age-standardized incidence rates with 95% confidence intervals of monthly cancer diagnoses for all-cancer in the Swiss cantons of Zurich and Zug stratified by incidence year (2018–2019); Table S1: Quasipoisson regression model estimates (with additional interaction term between incidence year and month) of age-standardized monthly incidence rates of all-cancer, colorectal cancer, female breast cancer, and prostate cancer diagnoses in the Swiss cantons of Zurich and Zug from 2018/19 to 2021.
