# Supplementary material for: Impact of the COVID-19 Pandemic and Lockdown on Cancer Diagnoses Using Swiss Cantonal Cancer Registry Data
Source: Cancers (Basel). 2024 Oct 3;16(19):3381. doi: 10.3390/cancers16193381 (PMC11482598; doi:10.3390/cancers16193381)
Supplement: Supplementary file 1 [file cancers-16-03381-s001.zip › cancers-3213693-supplementary.pdf]

Supplementary Material

Impact of the COVID-19 pandemic and lockdown on cancer diagnoses using Swiss cantonal cancer registry data

Authors

Flurina Suter[1,2], Miriam Wanner[2], Dominik Menges[1], Andreas Wicki[3], Dimitri Korol[2], Sabine Rohrmann[1,2,*]

Affiliation

[1] Division of Chronic Disease Epidemiology, Department of Epidemiology, Epidemiology, Biostatistics and Prevention Institute (EBPI), University of Zurich, Zurich, Switzerland

[2] Cancer Registry Zurich, Zug, Schaffhausen and Schwyz, Institute of Pathology and Molecular Pathology, University Hospital Zurich, Zurich, Switzerland

[3] Department of Medical Oncology and Hematology, Faculty of Medicine, University and University Hospital Zurich, Zurich, Switzerland.

*Correspondence: sabine.rohrmann@uzh.ch; Tel.: +41-446-345-256

Table of contents

Table S1: Quasipoisson regression model estimates (with additional interaction term between incidence year and month) of age-standardized monthly incidence rates of all-cancer, colorectal cancer, female breast cancer, and prostate cancer diagnoses in the Swiss cantons of Zurich and Zug from 2018/19 to 2021. 3

Figure S1: Absolute number of all-cancer cases stratified by incidence year, incidence month, and sex of the canton of Zurich from 2018/19 to 2021. 5

Figure S2: Absolute number of all-cancer cases stratified by incidence year, incidence month, and sex of the canton of Zug from 2018/19 to 2021. 6

Figure S3: Age-standardized incidence rates with 95% confidence intervals of monthly cancer diagnoses for colorectal cancer in the Swiss cantons of Zurich and Zug stratified by incidence year. 7

Figure S4: Age-standardized incidence rates with 95% confidence intervals of monthly cancer diagnoses for lung cancer in the Swiss cantons of Zurich and Zug stratified by incidence year. 8

Figure S5: Age-standardized incidence rates with 95% confidence intervals of monthly cancer diagnoses for skin melanoma in the Swiss cantons of Zurich and Zug stratified by incidence year. 9

Figure S6: Age-standardized incidence rates with 95% confidence intervals of monthly cancer diagnoses for female breast cancer in the Swiss cantons of Zurich and Zug stratified by incidence year. 10

Figure S7: Age-standardized incidence rates with 95% confidence intervals of monthly cancer diagnoses for prostate cancer in the Swiss cantons of Zurich and Zug stratified by incidence year. 11

Figure S8: Age-standardized incidence rates with 95% confidence intervals of monthly cancer diagnoses for all-cancer in the Swiss cantons of Zurich and Zug stratified by incidence year (2018-2019). 12

References: 13

Table S1: Quasipoisson regression model estimates (with additional interaction term between incidence year and month) of age-standardized monthly incidence rates of all-cancer, colorectal cancer, female breast cancer, and prostate cancer diagnoses in the Swiss cantons of Zurich and Zug from 2018/19 to 2021.

	All-Cancer[a,b] (n = 34,614)		Colorectal Cancer[a,b] (n = 3291)		Female Breast Cancer[a,c] (n = 4887)		Prostate Cancer[a,c] (n = 5474)	
	**Estimate**	**95% CI**	**Estimate**	**95% CI**	**Estimate**	**95% CI**	**Estimate**	**95% CI**
**Sex**								
Male (ref.)	1.00	-	1.00	-	-	-	-	-
Female	0.83	0.82-0.83	0.82	0.80-0.83	-	-	-	-
**Canton**								
Zurich (ref.)	1.00	-	1.00	-	1.00	-	1.00	-
Zug	0.95	0.95-0.96	1.28	1.25-1.32	1.13	1.10-1.16	0.91	0.89-0.93
**Incidence year**								
2018-2019 (ref.)	1.00	-	1.00	-	1.00	-	1.00	-
2020	1.02	1.01-1.03	1.21	1.12-1.32	0.74	0.67-0.82	1.18	1.06-1.30
2021	1.01	1.00-1.02	1.04	0.95-1.14	0.84	0.77-0.93	1.17	1.07-1.29
**Incidence month**								
January (ref.)	1.00	-	1.00	-	1.00	-	1.00	-
February	0.92	0.91-0.92	1.09	1.01-1.18	0.69	0.63-0.76	0.81	0.73-0.90
March	1.08	1.07-1.09	1.25	1.15-1.35	1.03	0.94-1.12	0.93	0.84-1.03
April	0.98	0.97-0.99	1.05	0.97-1.14	0.80	0.73-0.88	0.84	0.76-0.93
May	1.02	1.01-1.03	0.99	0.90-1.07	1.00	0.92-1.09	1.10	1.00-1.21
June	1.01	1.00-1.02	1.14	1.05-1.23	0.82	0.75-0.89	0.86	0.78-0.95
July	1.01	1.01-1.02	1.15	1.06-1.25	0.95	0.87-1.04	0.67	0.60-0.74
August	0.90	0.89-0.91	1.10	1.02-1.19	0.73	0.66-0.80	0.72	0.65-0.80
September	0.96	0.95-0.96	1.11	1.03-1.21	0.92	0.84-1.01	0.75	0.68-0.83
October	1.02	1.01-1.03	1.03	0.94-1.11	1.02	0.94-1.11	0.70	0.63-0.78
November	1.10	1.09-1.11	1.16	1.07-1.26	0.82	0.75-0.90	1.02	0.93-1.12
December	0.93	0.92-0.94	0.97	0.89-1.06	0.78	0.71-0.86	0.65	0.58-0.73
**Interaction year:month[d]**								
2020:February	1.03	1.01-1.04	0.92	0.82-1.04	1.55	1.34-1.80	0.78	0.67-0.91
2021:February	1.06	1.04-1.07	0.94	0.83-1.07	2.09	1.83-2.40	1.03	0.89-1.20
2020:March	0.93	0.92-0.94	0.70	0.62-0.79	0.93	0.80-1.07	1.07	0.93-1.23
2021:March	1.10	1.09-1.12	1.18	1.05-1.32	1.06	0.93-1.21	1.21	1.06-1.39

2020:April	0.70	0.69-0.71	0.49	0.42-0.57	0.95	0.81-1.11	0.48	0.40-0.57
2021:April	0.98	0.97-1.00	0.98	0.86-1.11	1.25	1.08-1.43	1.03	0.90-1.18
2020:May	0.85	0.83-0.86	0.88	0.78-0.99	0.96	0.83-1.11	0.60	0.52-0.69
2021:May	0.88	0.87-0.90	1.02	0.90-1.16	0.93	0.81-1.06	0.62	0.53-0.71
2020:June	0.93	0.92-0.94	0.64	0.56-0.72	1.60	1.39-1.84	0.77	0.67-0.90
2021:June	1.12	1.11-1.14	1.23	1.09-1.38	1.25	1.08-1.44	1.47	1.29-1.69
2020:July	0.97	0.96-0.99	0.94	0.84-1.05	1.28	1.12-1.47	0.97	0.82-1.14
2021:July	0.94	0.93-0.95	0.84	0.74-0.95	1.10	0.96-1.26	0.95	0.81-1.11
2020:August	1.01	0.99-1.02	0.73	0.64-0.82	1.23	1.06-1.43	0.86	0.74-1.01
2021:August	1.04	1.03-1.06	0.84	0.74-0.95	1.07	0.92-1.24	1.21	1.05-1.40
2020:September	1.07	1.05-1.08	0.89	0.79-1.00	1.05	0.90-1.21	0.88	0.75-1.03
2021:September	1.04	1.03-1.06	0.91	0.81-1.03	1.20	1.05-1.39	0.98	0.85-1.13
2020:October	0.92	0.91-0.93	0.83	0.74-0.94	1.30	1.13-1.48	0.86	0.73-1.01
2021:October	0.90	0.88-0.91	0.82	0.72-0.94	0.81	0.71-0.94	0.99	0.85-1.15
2020:November	0.94	0.93-0.95	0.67	0.59-0.76	1.80	1.57-2.07	0.87	0.76-1.00
2021:November	0.97	0.95-0.98	0.96	0.85-1.08	1.58	1.38-1.80	0.87	0.76-0.99
2020:December	0.99	0.98-1.00	1.00	0.88-1.13	1.54	1.34-1.78	1.14	0.98-1.33
2021:December	1.04	1.02-1.05	0.91	0.79-1.04	1.21	1.05-1.40	1.41	1.21-1.63

[a] Cancer cases were defined using the 10th revision of the international classification of diseases (ICD-10): All-cancer: all malignant cancers (except C44) and benign brain cancer (ICD-10: D32-33, D43); Colorectal cancer: ICD-10 C18-C20; Female breast cancer: ICD-10 C50; Prostate cancer: ICD-10 C61 [1].

[b] The Quasipoisson regression model was adjusted for canton (Zurich, Zug), sex (males, females), incidence year (2018-2021), incidence month (January-December), and the interaction between incidence year:incidence month.

[c] The Quasipoisson regression model was adjusted for canton (Zurich, Zug), incidence year (2018-2021), and incidence month (January-December), and the interaction between incidence year:incidence month.

[d] For the interaction term incidence year:month the reference categories were 2018/2019 (year) and January (month).

Figure S1: Absolute number of all-cancer cases stratified by incidence year, incidence month, and sex of the canton of Zurich from 2018/19 to 2021.

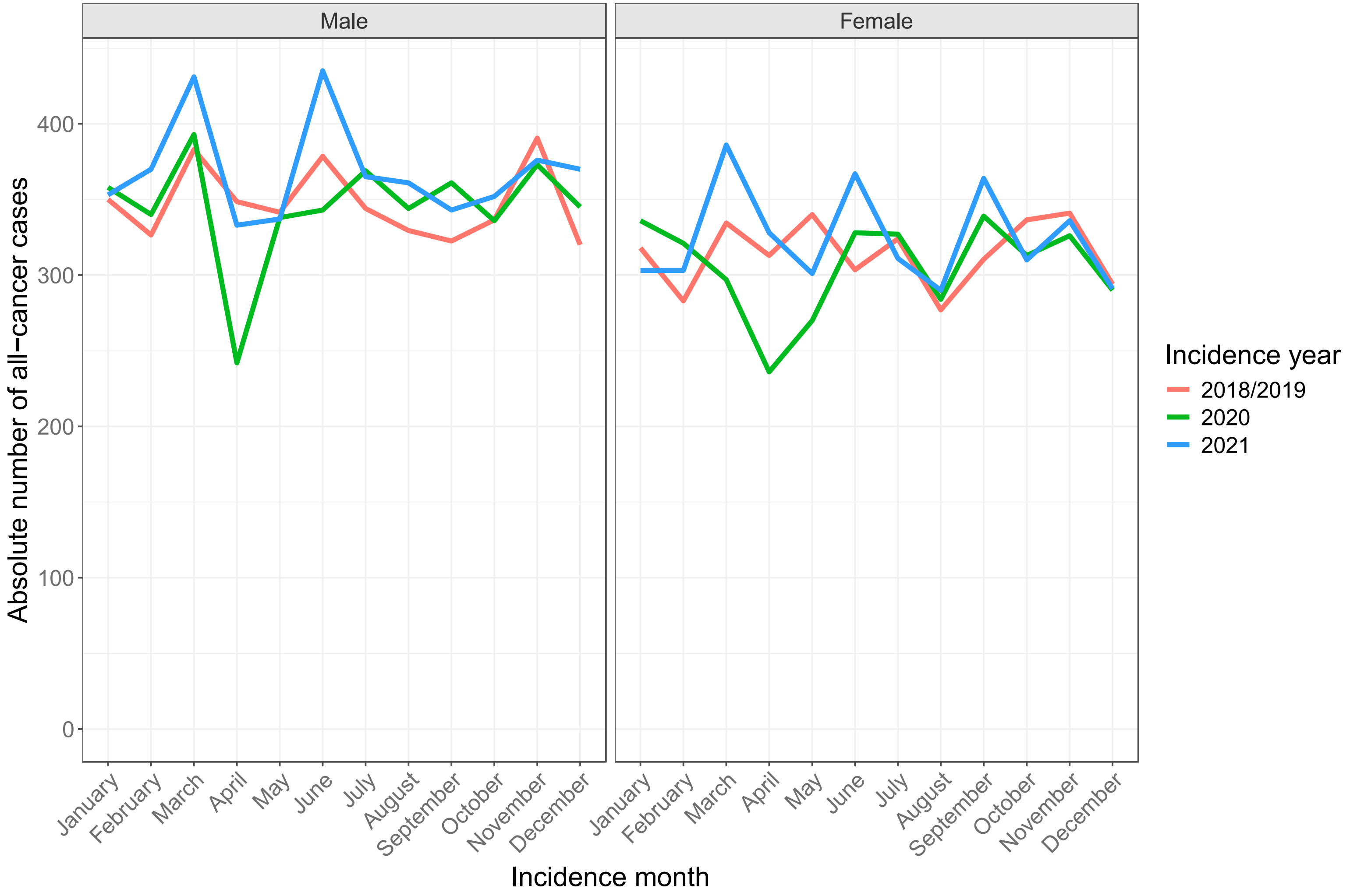

Figure S2: Absolute number of all-cancer cases stratified by incidence year, incidence month, and sex of the canton of Zug from 2018/19 to 2021.

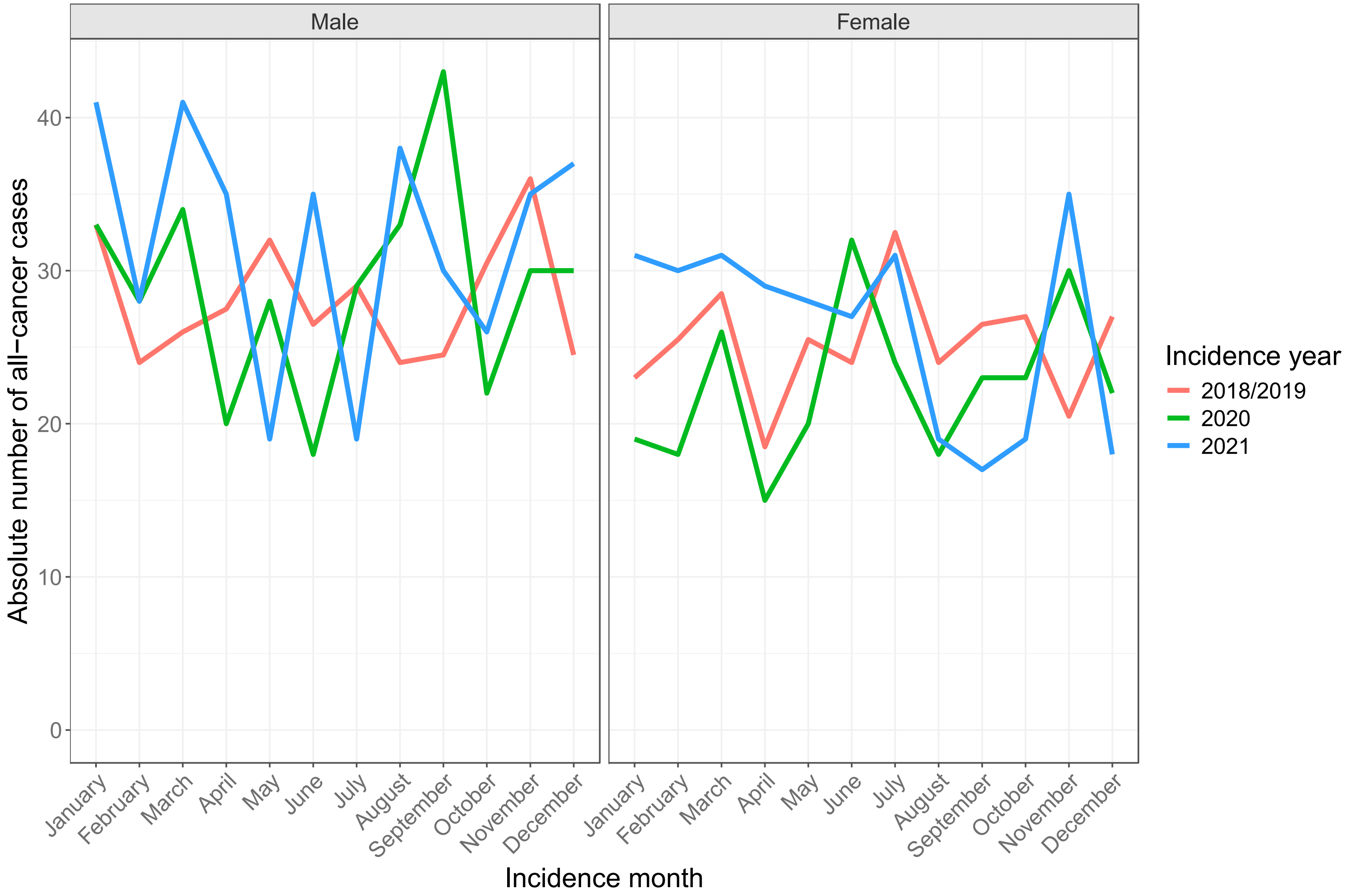

Figure S3: Age-standardized incidence rates with 95% confidence intervals of monthly cancer diagnoses for colorectal cancer in the Swiss cantons of Zurich and Zug stratified by incidence year.

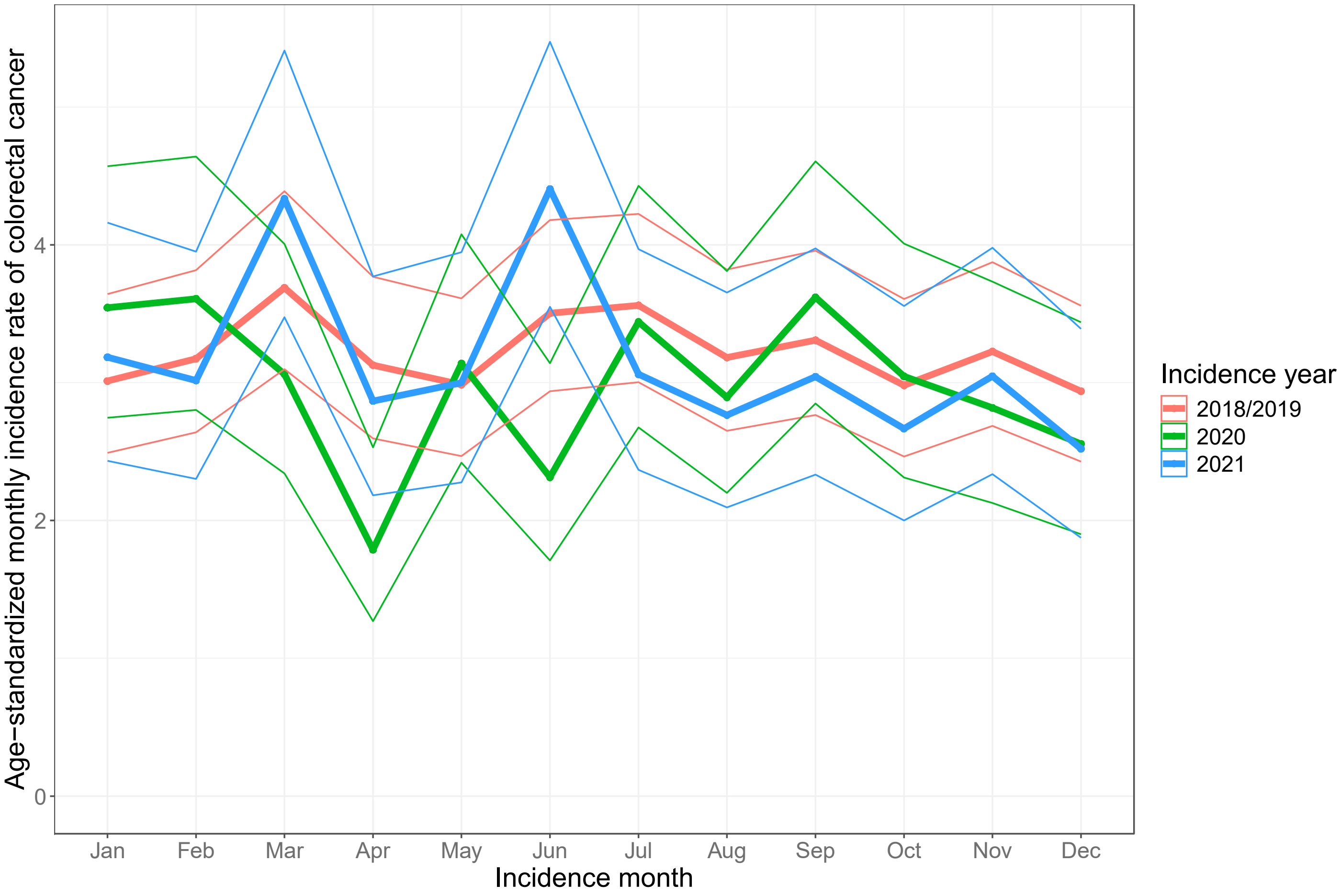

Figure S4: Age-standardized incidence rates with 95% confidence intervals of monthly cancer diagnoses for lung cancer in the Swiss cantons of Zurich and Zug stratified by incidence year.

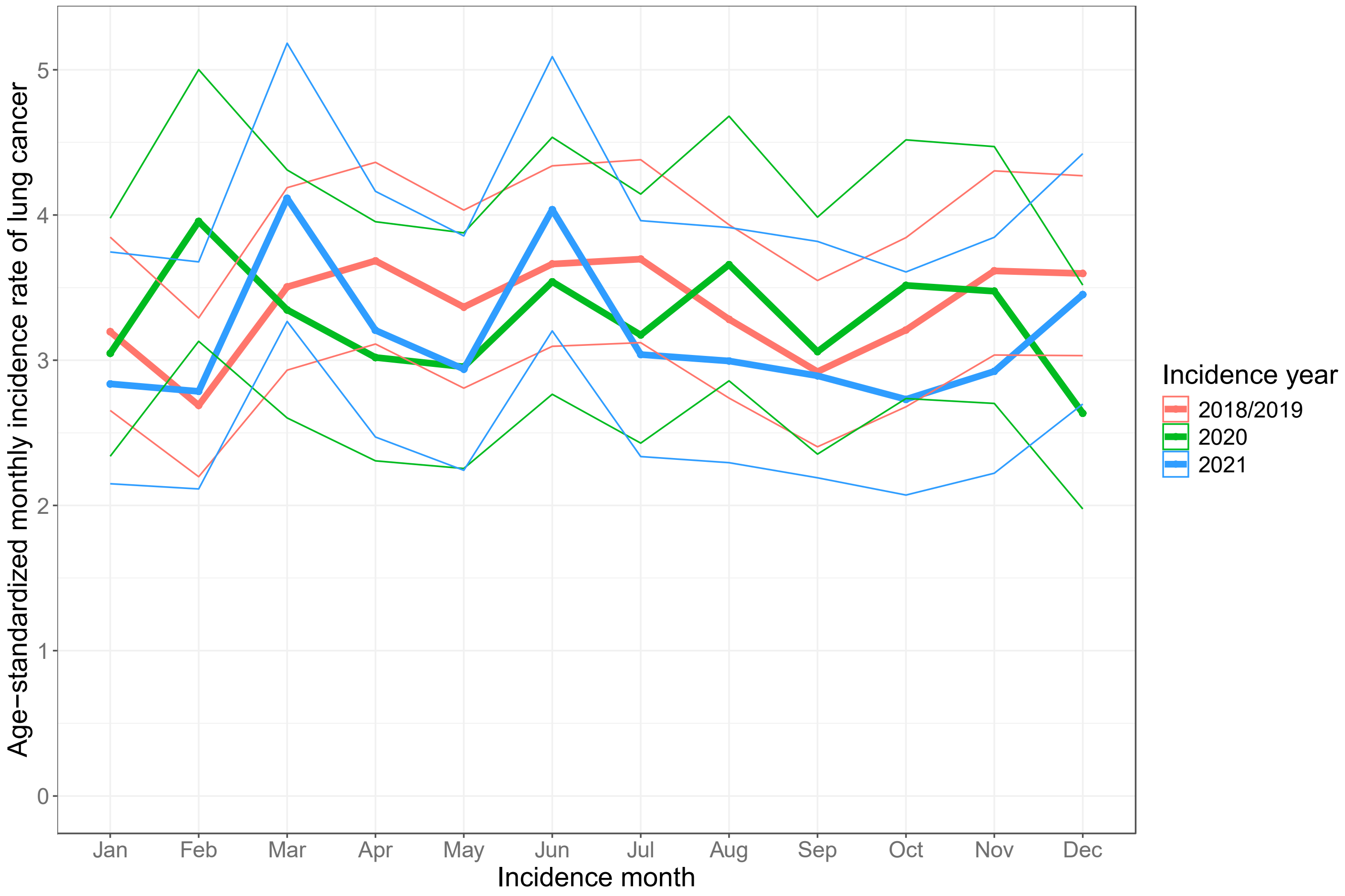

Figure S5: Age-standardized incidence rates with 95% confidence intervals of monthly cancer diagnoses for skin melanoma in the Swiss cantons of Zurich and Zug stratified by incidence year.

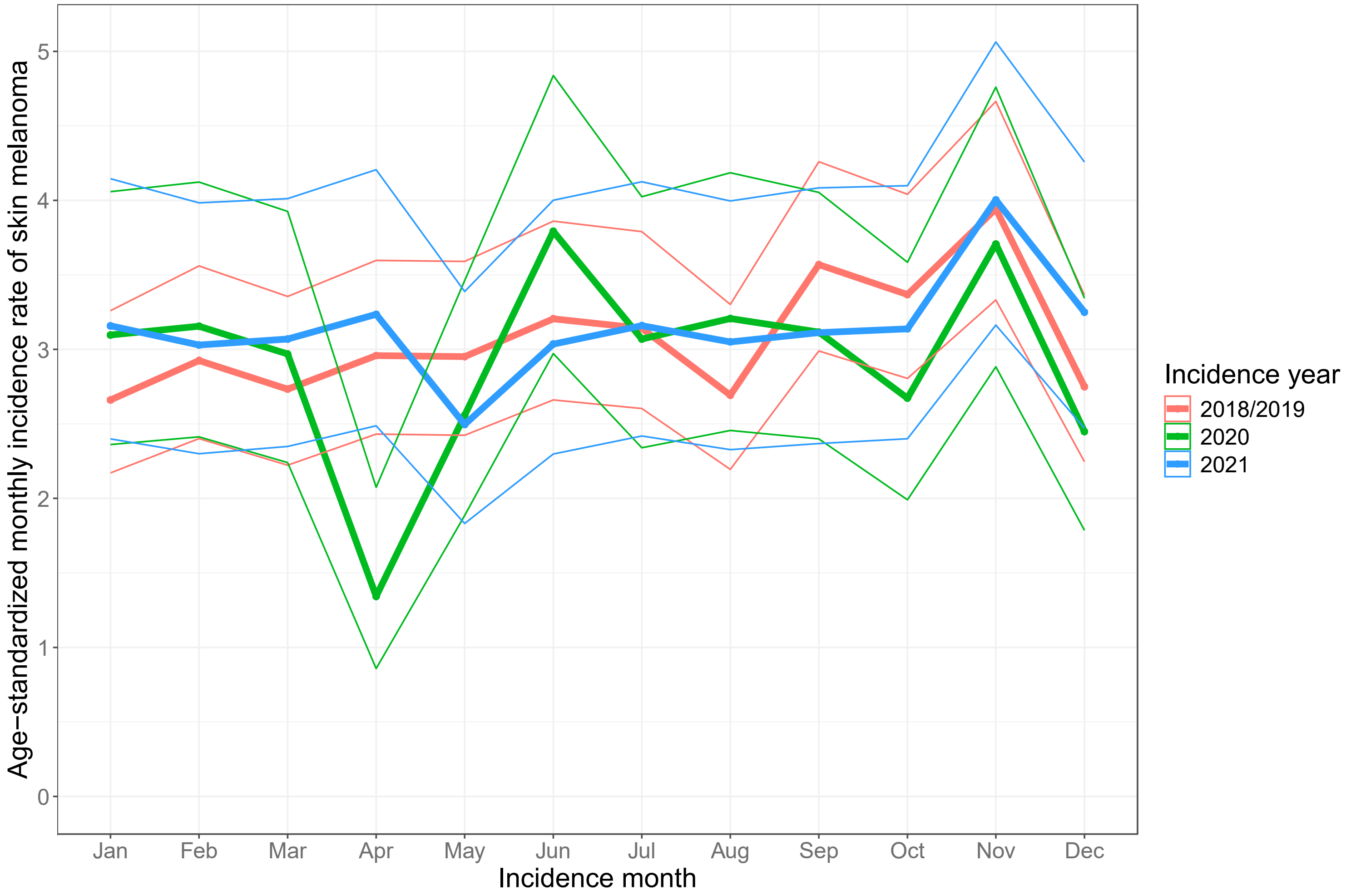

Figure S6: Age-standardized incidence rates with 95% confidence intervals of monthly cancer diagnoses for female breast cancer in the Swiss cantons of Zurich and Zug stratified by incidence year.

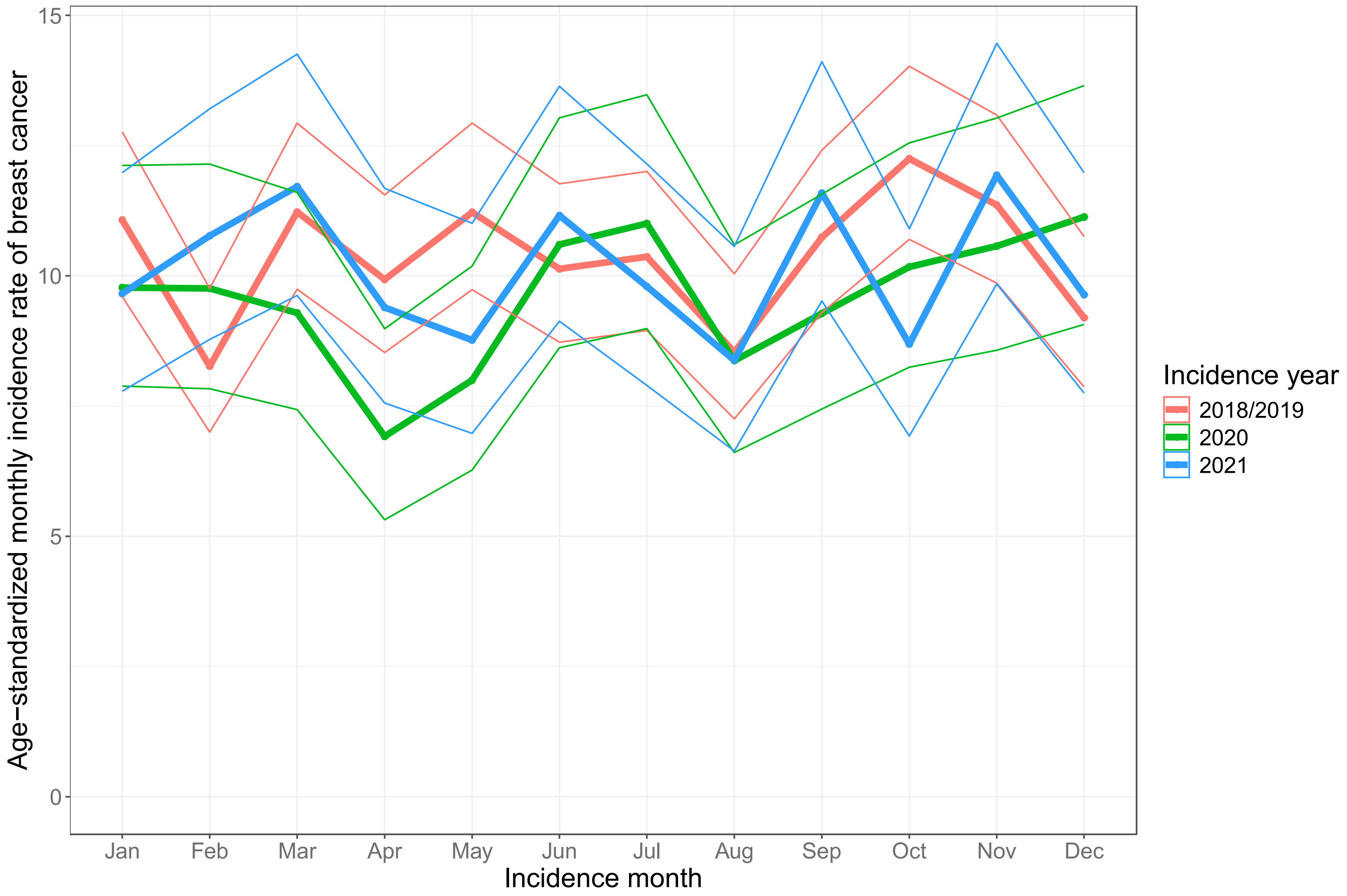

Figure S7: Age-standardized incidence rates with 95% confidence intervals of monthly cancer diagnoses for prostate cancer in the Swiss cantons of Zurich and Zug stratified by incidence year.

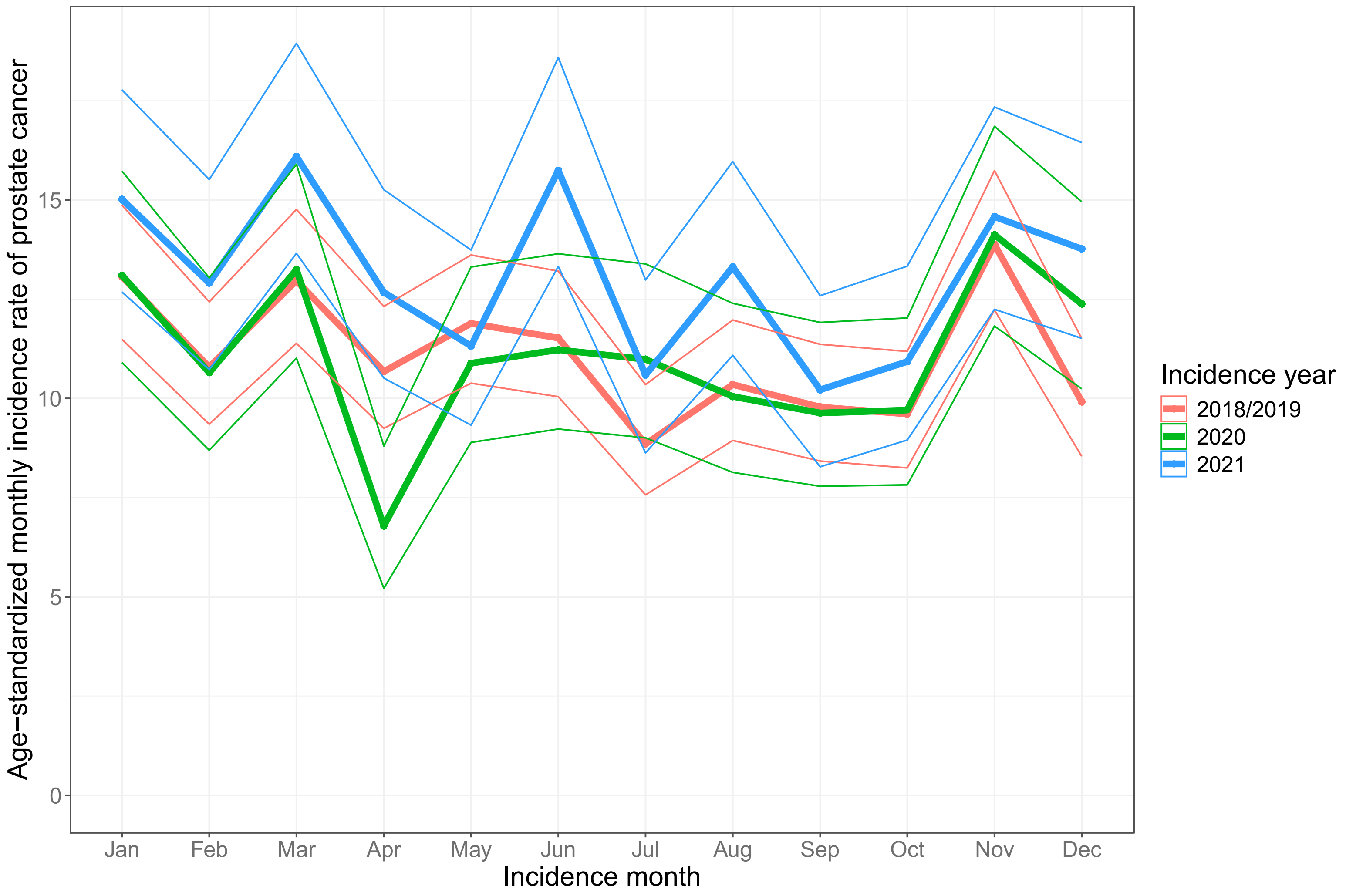

Figure S8: Age-standardized incidence rates with 95% confidence intervals of monthly cancer diagnoses for all-cancer in the Swiss cantons of Zurich and Zug stratified by incidence year (2018-2019).

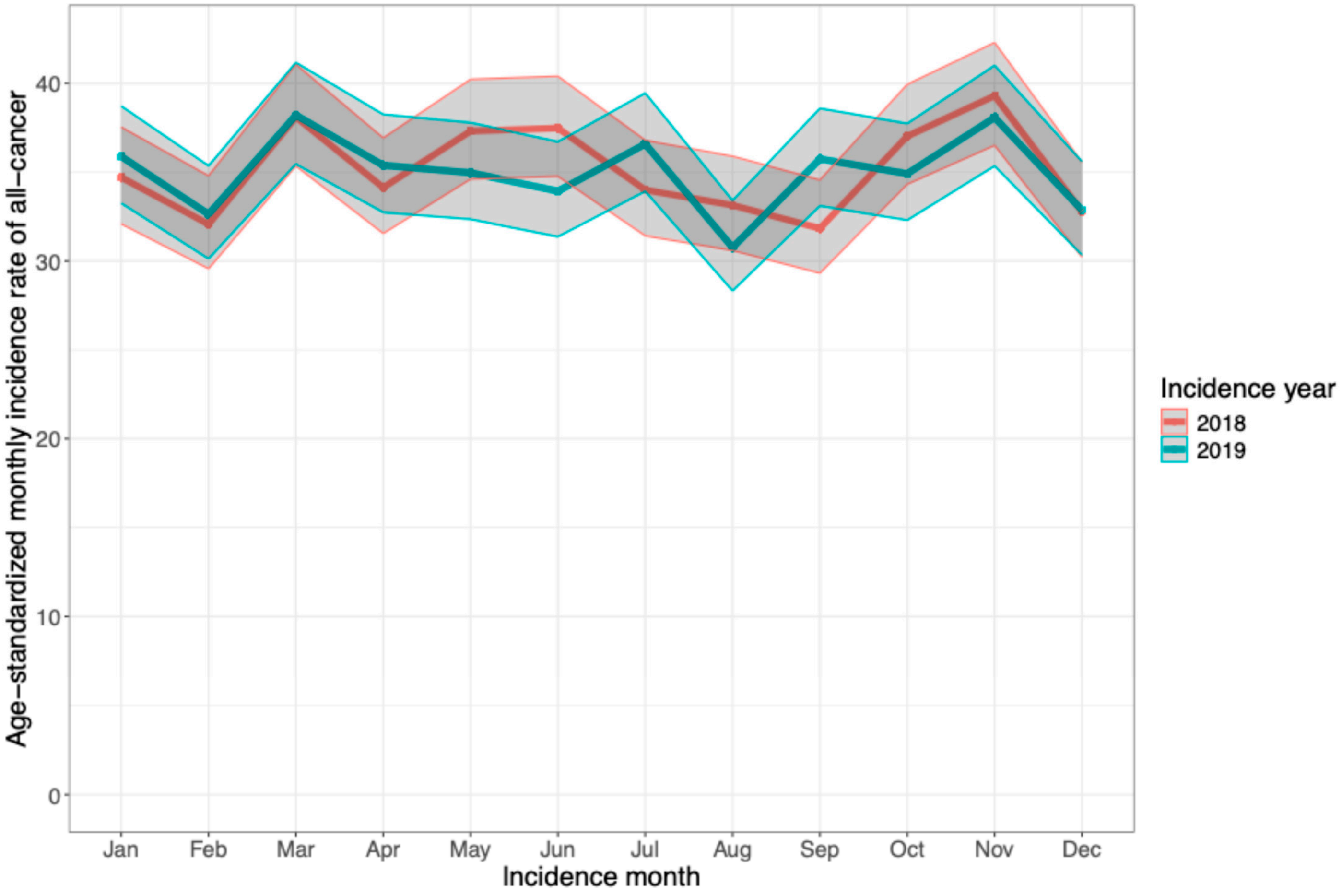

References:

1. World Health Organization. ICD-10 version: 2019 - International statistical classification of diseases and related health problems 10th revision. 2019. Accessed November 18, 2022. https://icd.who.int/browse10/2019/en
